# P-621. Metabolomic and Clinical Feature Analyses of Plasma from Influenza A Patients

**DOI:** 10.1093/ofid/ofaf695.834

**Published:** 2026-01-11

**Authors:** Yaping Li, Ting Li, Min Liu, Xinyu Wang, Song Zhai, Ruihong Qin, Yuanyi Chen, Chenrui Liu, Meng Zhang, Xiaoli Jia

**Affiliations:** Xi'an Jiaotong University Second Affiliated Hospital, Xi'an, Shaanxi, China; The Second Affiliated Hospital of Xi'an Jiaotong University, Xi'an, Shaanxi, China; The Second Affiliated Hospital of Xi'an Jiaotong University, Xi'an, Shaanxi, China; The Second Affiliated Hospital of Xi'an Jiaotong University, Xi'an, Shaanxi, China; Second Hospital of Xi'an Jiaotong University, Xi'an, Shaanxi, China; The Second Affiliated Hospital of Xi'an Jiaotong University, Xi'an, Shaanxi, China; The Second Affiliated Hospital of Xi'an Jiaotong University, Xi'an, Shaanxi, China; Xi'an Jiaotong University Second Affiliated Hospital, Xi'an, Shaanxi, China; The Second Affiliated Hospital of Xi'an Jiaotong University, Xi'an, Shaanxi, China; Xi'an Jiaotong University Second Affiliated Hospital, Xi'an, Shaanxi, China

## Abstract

**Background:**

Plasma metabolomics offers valuable insights for identifying viral infection biomarkers with applications in early diagnosis, outcome prediction, and treatment monitoring. This study aimed to investigate metabolomic alterations in influenza A patients and identify potential biomarkers for disease severity.

**Methods:**

From March 2023 to March 2024, 339 laboratory-confirmed influenza A patients (301 mild, 24 severe, and 14 critical cases) were enrolled from the fever clinic of the Second Affiliated Hospital of Xi’an Jiaotong University. After age‒sex matching, 54 patients (20 mild, 20 severe, and 14 critical cases) and 20 healthy controls were selected for analysis. Untargeted metabolomic profiling of 74 plasma samples was conducted using ultrahigh-performance liquid chromatography coupled with tandem mass spectrometry (UHPLC-MS/MS) with a Q Exactive™ Hybrid Quadrupole-Orbitrap mass spectrometer.

**Results:**

Comparative analysis revealed 60 differentially expressed metabolites between H1N1 patients and healthy controls, with 41 significantly upregulated and 19 downregulated. Pathway enrichment analysis revealed prominent disruptions in glycerophospholipid metabolism, with several metabolites showing alterations across the severity spectrum. Amino acid metabolism, particularly propionate metabolism, glycine-serine-threonine metabolism, and branched-chain amino acid biosynthesis, was also notably disturbed. Critical cases exhibited marked disturbances in taurine-hypotaurine metabolism compared to milder cases.

**Conclusion:**

This study identified glycerophospholipid metabolism dysregulation as a potential biomarker for influenza severity stratification. The progressive alteration of taurine pathway metabolites in critical cases suggests their pivotal role in severe H1N1 pathogenesis, highlighting their dual potential as diagnostic biomarkers and therapeutic targets.

**Disclosures:**

All Authors: No reported disclosures

